# Modulation of Mechanical Properties of Silica-Filled Silicone Rubber by Cross-Linked Network Structure

**DOI:** 10.3390/polym16162304

**Published:** 2024-08-15

**Authors:** Shuangyan Jiang, Zhanfu Yong

**Affiliations:** 1 School of Polymer Science and Engineering, Qingdao University of Science and Technology, Qingdao 266042, China; jsy42231814@163.com; 2 Qingdao Automotive Research Institute, Jilin University, Qingdao 266061, China

**Keywords:** silicone rubber, inhomogeneous cross-linked network, vinyl, mechanical properties

## Abstract

Associating molecular structure and mechanical properties is important for silicone rubber design. Although silicone rubbers are widely used due to their odourless, non-toxic, and high- and low-temperature resistance advantages, their application and development are still limited by their poor mechanical properties. The mechanical properties of silicone rubbers can be regulated by designing the cross-link density and cross-linking structure, and altering the molar contents of vinyl in the side groups of methyl vinyl silicone rubber (MVQ) leads to different cross-linking structures and cross-linking densities in the vulcanized rubber. Therefore, this study investigated the differences in molecular parameters and molecular chain structures of unprocessed MVQ rubbers with different vinyl contents. The results showed that MVQ rubbers with high vinyl contents were branched polymers, better facilitating the cross-linking reaction than MVQ rubbers with low vinyl contents. In addition, silicone rubbers with different vinyl contents were co-cross-linked to introduce an inhomogeneous cross-linked network in the silicone rubber to improve its mechanical properties. The cross-linked network properties were analysed by the Flory–Rehner model and Mooney–Rivlin plots, and it was found that the long chains in the sparsely cross-linked domains of the network favoured high elongation at break and the short chains in the densely cross-linked domains contributed to high modulus, which could satisfy the functions of reinforcing and toughening the rubber materials at the same time. It was also found by analysing the filler network and aggregate morphology that the inhomogeneous cross-linked network led to an improvement in the dispersion of silica in the rubber and a significant improvement in the mechanical properties of silicone rubber.

## 1. Introduction

With the constant advance of aerospace technologies, the requirements for sealing products are becoming increasingly demanding. Silicone rubbers have unique properties due to the silicone–oxygen bonds forming their main chains, which include excellent aging properties, low-temperature resistance, high-temperature resistance, low surface tension, and good biocompatibility [1,2,3]. With these unique properties, silicone rubbers have become irreplaceable in aerospace, military, and other applications. However, the intermolecular forces of silicone–oxygen bonds in silicone rubbers are weak due to their molecular structure. As a result, their mechanical properties, especially tear strength, are poor, limiting their applications. Therefore, improving the mechanical properties of silicone rubbers is crucial [4]. The poor mechanical properties of pure silicone rubbers usually necessitate adding various fillers for reinforcement in practical applications [5], such as silica, montmorillonite, and mica [6]. To some extent, these fillers improve the mechanical properties of silicone rubbers. Yet, the filler dispersion problem hinders further mechanical property improvements. Ke Cao et al. [7] used γ-ray irradiation of 3-methacryloxypropyltrimethoxy silane (MPTMS) to polymerize on the surface of GO and CNTs in order to improve interfacial interactions between GO/CNTs-Si and the SR matrix. The prepared GO/CNT-Si not only exhibited better dispersion but also improved the mechanical properties of SR. Rubber blending is an effective method to improve the mechanical properties of SR. Hui Li et al. [8] prepared fluorine/silicone composite rubbers by mechanical blending process using three different fluoroelastomer systems to find the optimal composites. Silicon dioxide and N990 as fillers have a synergistic effect, which is conducive to the combination of FKM and MVQ and improves the mechanical properties of the composites.

In addition, the mechanical properties depend on the cross-link density and overall rubber network structure [9,10]. An increased cross-link density of the elastic rubber network increases the strength but decreases the toughness. Unevenly cross-linked networks (densely and sparsely cross-linked domains) offer the opportunity to simultaneously strengthen and toughen the rubber material. The highly stretchable molecular chains in the densely cross-linked domains provide a higher modulus, while the molecular chains in the sparsely cross-linked domains are flexible and highly stretchable [11]. During deformation, the densely cross-linked domains in such an inhomogeneous cross-linked network structure preferentially rupture and dissipate a large amount of energy, thereby improving mechanical properties [12].

**Figure 1:**
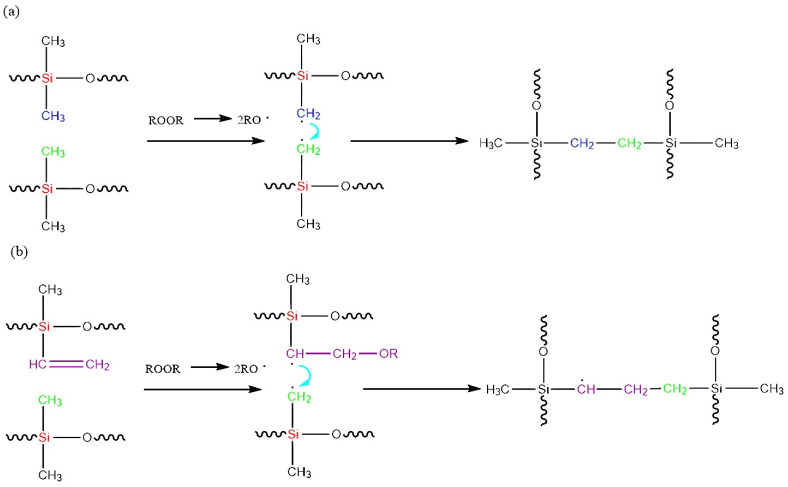
Reaction of methyl vinyl silicone rubber and DBPMH: (**a**) carbon atoms in two methyl groups of silicone rubber form a cross-linking bond and (**b**) carbon atoms in the double bond form a cross-linking bond with carbon atoms in the methyl group.

The silicone rubber network structure is formed through free radical reactions during cross-linking [13]. Methyl vinyl silicone rubber (MVQ) is one of the most commonly used silicone rubbers, usually initiated with organic peroxides to form a three-dimensional network structure through free radical reactions. In the presence of a cross-linking agent, two types of cross-linking reactions occur in silicone rubbers (Figure 1 shows the reaction between methyl vinyl silicone rubber and DBPMH): the formation of cross-linking bonds between carbon atoms in the two methyl groups of the silicone rubber and between carbon atoms in the double bonds and the methyl groups. Thus, methyl and vinyl are the major cross-linking reaction sites in MVQ [14]. The mechanical properties are affected greatly by the molar masses between cross-linking points (*M_c_*) and *M_c_* distribution. In particular, bimodal networks composed of long chains and short chains can result in enhanced mechanical properties, when the molar masses of long and short chains differ by over 10 times [15]. Wang et al. [16] investigated the evolution of cross-linking structure and changes in cross-linking density at different vinyl contents and pre-curing times in MVQ, and found that controlling the cross-linking density and structure by vinyl content was effective. Wei et al. [17] investigated the effect of cross-link density on the properties of silicone rubber and found that the elongation at break decreases with increasing cross-link density. By mixing silicone rubbers with different vinyl contents, Xu Qiang et al. [4] and Lu Gan et al. [13] found that the mechanical properties of the prepared rubber hybrids and their composites showed more significant improvements than silicone rubber composites with a single vinyl concentration. They prepared PDMS blends, which performed higher tear strengths and moduli than control ones. But tensile strengths and elongations at break were decreased. Besides, it seemed that the gaps between long and short chain lengths were all below 10 times than those in Xu’s samples. Therefore, it still has great potential to further improve the mechanical properties of silicone rubber by developing a certain structure of a cross-linking network.

Blending MVQ rubbers with different vinyl contents is a good solution for constructing inhomogeneous, cross-linked network structures. This study prepared MVQ gums with three vinyl contents to form densely and sparsely cross-linked structural domains. Meanwhile, a homogeneous cross-linked network was formulated with a single gum of the corresponding vinyl content to serve as a control. The effects of molecular structure, cross-linking structure, and cross-link density on the mechanical properties of MVQ rubbers with different vinyl contents were systematically discussed. The results revealed the contribution of the inhomogeneous cross-linked network structure to mechanical property improvement. In addition, the effect of vinyl content on the cross-link density and structure of MVQ rubber was investigated, and altering the vinyl content proved effective in controlling the cross-link density and structure. This study could contribute to a deeper understanding of the relationship between the cross-linking structure and the physicomechanical properties of silicone rubbers and the modulation of such properties by altering the cross-linked network.

## 2. Materials and Methods

### 2.1. Materials

MVQ rubbers with three different vinyl contents were provided by Hubei Xingrui Silicone Material Co., Ltd. (Yichang, China). The vinyl molar contents were 3% (grade 201 with a Mooney Viscosity of 4.0), 0.22% (grade 110-3 with a Mooney Viscosity of 3.9), and 0.08% (grade 110-1 with a Mooney Viscosity of 4.1). Fumed silica with a specific surface area of 200 m^2^·g^−1^ was manufactured by Evonik Degussa (China) Co., Ltd. (Shanghai, China). The hydroxyl silicone oil was purchased from Jiangsu Quanli Chemical Co., Ltd. The 2,5-Dimethyl-2,5-di(tert-butylperoxy)-hexane (DBPMH) was supplied by Shin-Etsu Chemical Co., Ltd. (Tokyo, Japan).

### 2.2. Gel Permeation Chromatography Characterization

The molecular weight (MW) and molecular weight distribution (MWD) of the silicone rubber were determined through gel permeation chromatography (GPC) using an HLC-8320 GPC system (Tosoh Bioscience, Shonan City, Japan). The mobile phase was tetrahydrofuran at a flow rate of 1.0 mL·min^−1^, and the testing temperature was 40 °C.

### 2.3. Sample Preparation

First, the initial temperature of the refiner was set at 135 °C, and the rotational speed was set at 60 rpm. After reaching the set temperature, VMQ rubber was put into the refiner for moulding for 2 min until the torque stabilized. The silica and hydroxyl silicone oil were divided into 3 parts, each added to the mixer for mixing until the torque stabilized (2 min) before adding the next part. The rubber was discharged from the mixer after the final torque stabilized. The blended rubber was allowed to cool to room temperature, and the vulcanizing agent DBPMH was added to the opener in several portions. The silicone rubber formulations are presented in Table 1. After standing for 24 h, the vulcanization curves of the rubbers were tested, and the vulcanization was carried out.

### 2.4. Vulcanization Characteristic Testing

The process positive vulcanization time t90 was measured on a rotorless vulcanizer (MDR), followed by vulcanization in a flatbed vulcanizing machine at t90 + 2 min and 170 °C. Peroxides generally require a second vulcanization stage (200 °C × 2 h). For one thing, it can improve the cross-linked network. For another, it removes excess peroxides and enhances product stability.

### 2.5. Cross-Link Density Testing

To assess the possible effect of the vinyl contents of MVQ blends on the cross-link density of vulcanized rubbers, the cross-link density was tested using the equilibrium swelling method. Approximately 2 to 3 g of vulcanized rubber weighing $m_{0}$ was immersed in an appropriate amount of toluene solution for 72 h. The immersed rubber material was removed, and its surface was wiped clean with toluene before weighing. Then, the material was weighed every 12 h until the mass stabilized. The final mass was recorded as $m_{1}$. The cross-link density was estimated using the Flory–Rehner model, as expressed in the following equation [18,19]:

(1) $$V_{r}=\frac{1}{1+\left(m_{0}/m_{1}-1\right)\times \rho /\rho_{s}\times \alpha }$$

where $V_{r}$ is the volume fraction of the rubber after swelling, ρ is the density of the rubber compound, and $\rho_{s}$ is the density of the toluene solvent, which is 0.872 $g/{cm}^{3}$.

(2) $$\upsilon_{c}=-\frac{\ln\left(1-V_{r}\right)+V_{r}+\chi V_{r}^{2}}{V_{s}\left(V_{r}^{1/3}-V_{r}\right)}$$

where $\upsilon_{c}$ is the cross-link density of the rubber composite, $V_{s}$ is the molar volume of the solvent toluene, which is 106.54 ${cm}^{3}/mol$, and χ is the Flory–Huggins parameter of the silicone rubber and toluene, which is 0.465.

### 2.6. Mechanical Properties Testing

The tensile force was measured on a GT-AT-7000M electronic tensile testing machine (Goodtechwill, Qingdao, China) at 500 mm/min using dumbbell-shaped samples, according to ISO 37:2024 [20]. Stress–strain curves were plotted for each sample. Tear tests were conducted under the same conditions, except that right-angle samples were used (ISO 34-1:2022 [21]). These tests measured the tensile strength, elongation at break, tear strength, and Young’s modulus. Hardness (Shore A) was measured using a Shore A hardness tester (MonTech Trading Co., Ltd., Tianjin, China), according to the ASTM D 2240-2005 [22] method.

### 2.7. Friction Testing

The coefficient of friction was tested using a friction tester (HEIDON, Shinto Scientific Co., Ltd., Tokyo, Japan) at a 100 mm/min speed, a 5 N load, and a 100 mm displacement. The vulcanized force sheet was cut into uniform sizes, mounted on the test area, and tested according to the set test conditions. The force vs. displacement curves were plotted, and the average values of the stabilized curves were taken as the friction force through graphing, which was the coefficient of friction by dividing it by the load force.

### 2.8. Rubber Process Analysis

A D-RPA 3000 dynamic rubber process analyser (MonTech Trading Co., Ltd., Tianjin, China) was used to measure the strain amplitude dependence of viscoelastic parameters in VMQ composites. The Payne effect test was conducted at 70 °C, under the strain range of 0.1% to 100% and the frequency of 1 Hz. The large amplitude oscillation shear (LAOS) test was conducted on raw rubber under a frequency of 1.0 Hz, a strain range of 0.1% to 1500%, and a temperature of 170 °C. Frequency scanning of vulcanized rubber was performed at 60 °C under the frequency range of 0.01 to 30 Hz and a strain of 7%.

### 2.9. Scanning Electron Microscopy Testing

To study the morphology and dispersion of silica in the rubber matrix, tensile sections of the composites were observed through scanning electron microscopy (SEM; JSM-7500F; Nippon Electronics Corporation, Tokyo, Japan).

## 3. Results and Discussion

### 3.1. Molecular Weight and Molecular Weight Distribution

**Figure 2:**
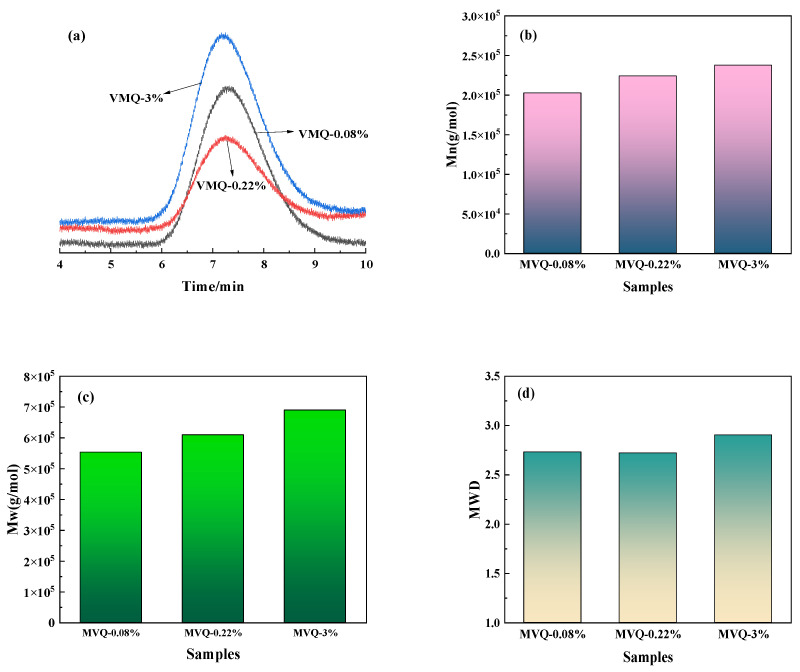
GPC curves for the molecular weight and molecular weight distribution of the three MVQ rubbers with different vinyl contents: (**a**) GPC curves, (**b**) number-average molecular weight (Mn), (**c**) weight-average molecular weight (Mw), and (**d**) molecular weight distribution (MWD).

The GPC curves of MW and MWD for the three MVQ rubbers with different vinyl contents are shown in Figure 2. The larger MW and wider MWD of MVQ-3% compared to MVQ-0.08% and MVQ-0.22% imply its stronger molecular interactions and higher raw rubber strength. In addition, MVQ-0.08% has a wider MWD compared to MVQ-0.22%, suggesting its better processing performance than MVQ-0.22%. Considering the small differences in Mn, Mw, and MWD, this paper delved primarily into the effect of vinyl content on the mechanical properties and cross-linking properties of vulcanized MVQ rubbers, ignoring the effects of MW and MWD of MVQ rubbers with different vinyl contents.

### 3.2. LAOS Analysis

**Figure 3:**
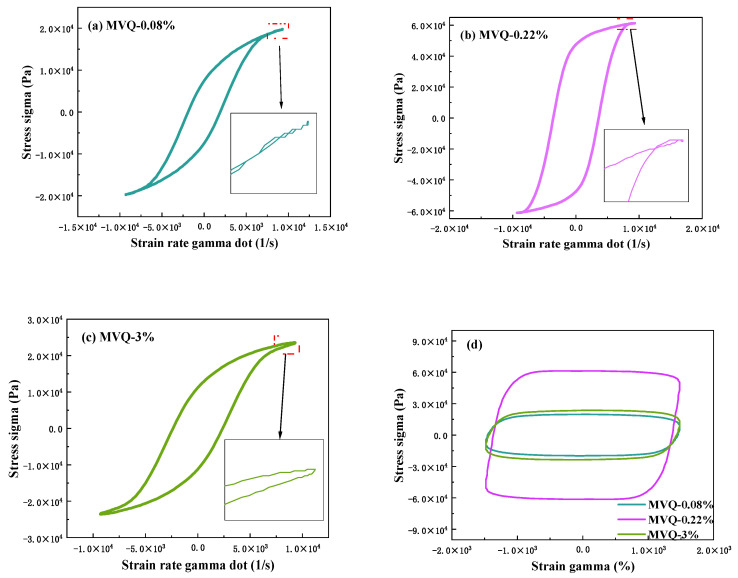
Lissajous curves of raw MVQ rubbers with three different vinyl contents: (**a**) viscous Lissajous curve for MVQ-0.08%, (**b**) viscous Lissajous curve for MVQ-0.22%, (**c**) viscous Lissajous curve for MVQ-3%, and (**d**) elastic Lissajous curve.

In addition to composition, MW, and MWD, another key parameter controlling polymer performance is the degree of branching [23]. The LAOS test is sensitive to long-chain branching and is a useful characterization tool [24]. Silicon–oxygen bonds form the main chain of silicone rubbers, while the methyl and vinyl groups on the side chains are the main cross-linking reaction sites in MVQ rubbers. Thus, the degree of branching of MVQ rubbers also affects the cross-linking results and cross-link density. Figure 3 shows the Lissajous curves of the raw rubbers with three different vinyl contents under large strains. The presence of secondary rings in the Lissajous curves [25] is related to the polymer’s molecular structure (i.e., linear, star, and branched). Linear polymers usually exhibit secondary double rings, while branched polymers do not. As shown in Figure 3a,b, the presence of secondary double rings at both ends of the curves suggests that the MVQ rubber with a low vinyl content is linear. In contrast, the curve in Figure 3c shows no secondary double rings, confirming that MVQ rubber with high vinyl content is a branched polymer.

The LAOS test can quantify the long chain branching (LCB) index, with a higher LCB index indicating a higher degree of branching. A negative LCB index indicates that the polymer is a linear polymer, whereas branched polymers have a positive LCB index. Table 2 lists the LCB indexes of MVQ rubbers with different vinyl contents, and the negative LCB indexes confirm the linearity of VMQ-0.08% and VMQ-0.22%. It can also be concluded that the LCB index increases with increasing molecular weight and that more branched chains are present at high molecular weights, as previously reported by Suárez et al. [26]. The increased vinyl content provides cross-linking points for the MVQ rubber, making it easier to quickly form a three-dimensional network structure and limiting the movement of the molecular chains.

### 3.3. Vulcanization Properties

**Figure 4:**
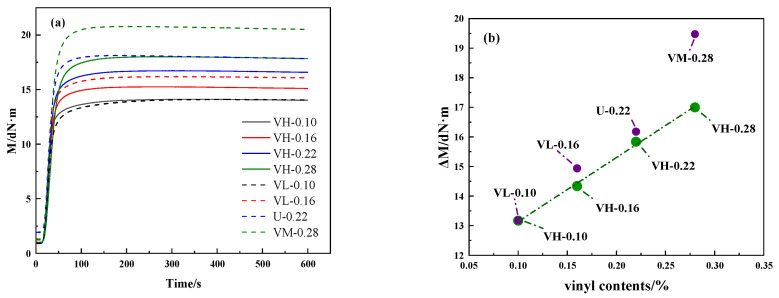
(**a**) Vulcanization curve of MVQ composites and (**b**) vinyl content versus effective torque curve.

Figure 4a shows the torque curves of the composites prepared by blending MVQ rubbers with different vinyl contents during vulcanization. From Figure 4a, it can be learnt that the maximum torque value increases as the total vinyl content increases. The effective torque (ΔM = M_H_ − M_L_) is the difference between the maximum torque (M_H_) and the minimum torque (M_L_) during vulcanization, which is usually considered to be related to the cross-link density of the rubber network [5]. The vinyl content versus ΔM curve is shown in Figure 4b, where the cross-link density of the composites increases with the vinyl content. Meanwhile, the VH samples have the lowest ΔM overall, mainly because the vinyl dispersion differences under the same vinyl content cause the samples to exhibit different cross-linking states. In turn, the samples show different molecular weights between cross-linking points, forming densely and sparsely cross-linked domains, exhibiting cross-link density differences.

### 3.4. Network Structure Analysis

The $\upsilon_{e}$ values of vulcanized rubbers prepared by blending MVQ rubbers with different vinyl contents were measured using the equilibrium swelling method (Table 3). $\upsilon_{e}$ increases with the vinyl content, which agrees with the ΔM results. The VH samples have lower $\upsilon_{e}$ values under the same vinyl content. With tighter cross-linking, the molecular chain segments between the cross-linking points shorten, and the flexibility of the molecular network decreases, rendering it difficult for solvent molecules to enter this rigid molecular network. However, the increased cross-linking points are locally concentrated in the VH samples, and densely cross-linked domains are present with small percentages. Thus, their solvent-carrying capacity is less affected, and the long chains in the sparsely cross-linked domains still dominate, lowering the cross-link density.

**Figure 5:**
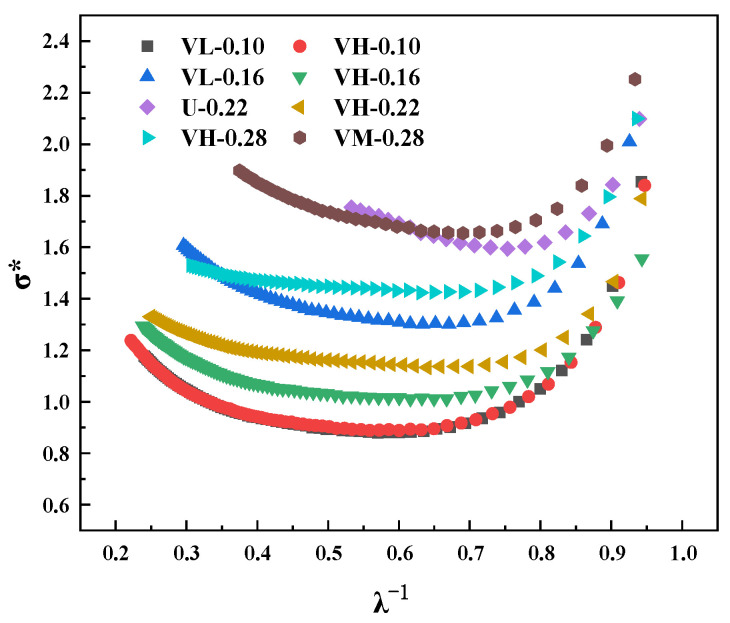
Mooney–Rivlin curves for the composites.

In addition, the Mooney–Rivlin model is used to evaluate the cross-link density, as expressed in Equations (3) and (4) [27,28]. Figure 5 shows the Mooney–Rivlin curves of the composites obtained by blending MVQ rubbers with different vinyl contents.

(3) $$\sigma^{*}=\frac{\sigma }{\left(\lambda -\lambda^{-2}\right)}=2\left(C_{1}+\frac{C_{2}}{\lambda }\right)$$

where σ* is the reduced stress, *σ* is the true stress, and *λ* is the elongation. *C*_1_ and *C*_2_ are constants determined by the intercept and slope of the linear region in Figure 5, respectively. Constant *C*_1_ is related to the cross-link density $\upsilon_{c}$, as expressed below:

(4) $$C_{1}=\frac{\rho RT}{2M_{c}}=\upsilon_{c}RT$$

where R is the gas constant (8.314 J·mol^−1^·K^−1^), T is the absolute temperature (298 K), ρ is the density of the rubber, and $M_{c}$ is the number-averaged molecular weight between adjacent cross-linking bonds.

As shown in Figure 5, the Mooney–Rivlin diagram includes the low-strain (1/*λ* > 0.9), medium-strain, and high-strain (1/*λ* < 0.6) regions [19]. In the low-strain region, the σ* stress decreases with the increasing strain, possibly because the rubber network deformation leads to molecular entanglement slippage, reducing the contribution of the entanglements [29]. In the high-strain regions, the composites also show an upward curvature as the strain increases, which can be attributed to the non-Gaussian nature of the molecular network [30]. The $\upsilon_{c}$ values of the composites based on the equilibrium swelling method are compared with those estimated using the Mooney–Rivlin model (Table 3). The trend of $\upsilon_{c}$ estimated based on the C_1_ value is consistent with that obtained through the equilibrium swelling method. Compared to the VH samples, the rise in σ* stress starts at low strains in the other samples, which can be ascribed to the increase in shorter network chains [31]. In the VH samples, the inhomogeneous cross-linked network has a smaller proportion of densely cross-linked domains, while the long chains in the sparsely cross-linked domains still dominate.

### 3.5. Mechanical Properties Analysis

**Figure 6:**
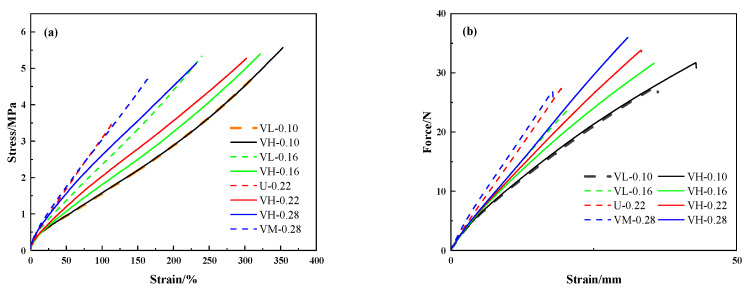
(**a**) Stress–strain curve and (**b**) displacement versus tearing force curve.

The mechanical properties of the samples are presented in Table 4, and the stress–strain curves of the silicone rubbers are shown in Figure 6a. Table 4 and Figure 6a show that when MVQ rubbers with different vinyl contents are blended, the vinyl groups are not uniformly distributed, giving them better tensile properties. In terms of tearing properties (Figure 6b), the VH samples had higher tear strengths, which increased with the total vinyl content. These property differences are due to the effect of inhomogeneous cross-linking. The cross-linked network was inhomogeneously cross-linked in samples prepared by blending MVQ rubbers with different vinyl contents, whereas the rubbers prepared with a single vinyl content had evenly cross-linked networks.

**Figure 7:**
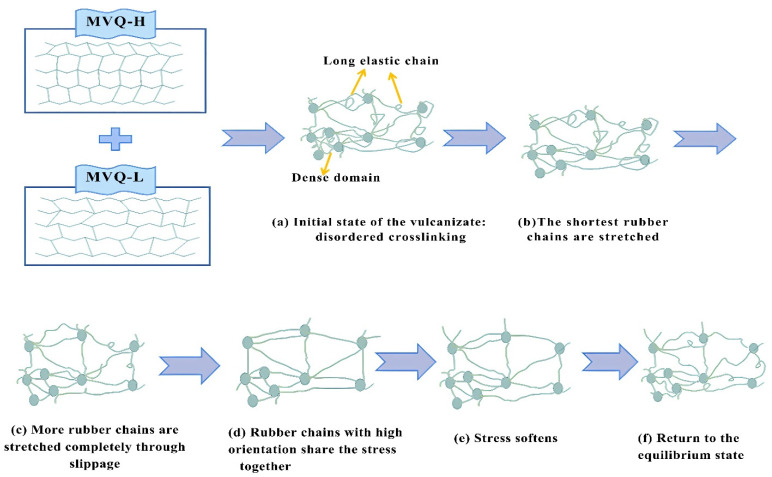
Slippage and parallel arrangement of elastomer molecular chains in the stretching state.

The molecular chain movement of the inhomogeneous cross-linked network in the stretched state is shown in Figure 7. With more vinyl groups, the MVQ rubber has more cross-linking points within the molecular chain. Thus, after vulcanization, these cross-linking points in the MVQ rubber can share more tensile force during stretching [32], mainly because the short chains in the densely cross-linked domains promote stress dispersion. In addition, MVQ rubbers can also be made more elastic due to the presence of uneven cross-linking points. The densely cross-linked domains dispersed in the rubber network act like islands, making the long chains in the base network more ductile, reaching higher elongation at break.

**Figure 8:**
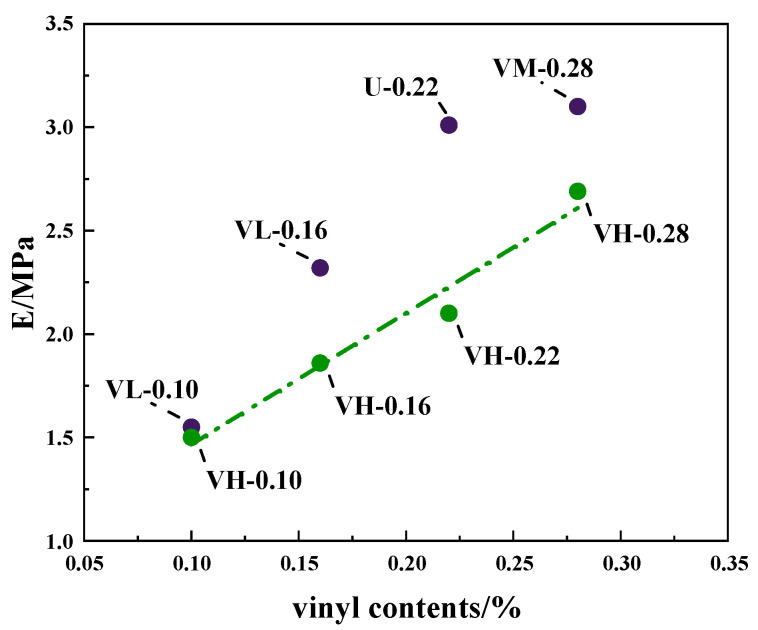
Young’s modulus (E) versus vinyl content curves of composites.

The increased cross-link density improves the modulus. Generally, a high cross-link density tends to cause chain segment breakage, restrict chain segment movements, and reduce the average molecular weight (*M_c_*) between cross-links, thereby reducing flexibility [33]. In the VH samples, the stress can be easily dispersed under increased cross-link density. As a result, the modulus of the MVQ rubber is improved. Figure 8 shows Young’s modulus (E) of the composites versus vinyl content, where the modulus of the VH samples increases as the vinyl content (cross-link density) increases. In addition, the mechanical properties (including elasticity) of the silicone rubber show no significant decrease, which can be attributed to the inhomogeneous cross-linked network of the VH samples. The short chains in the densely cross-linked domains (Figure 7a) promote stress dispersion, thus increasing their modulus. In addition, the long chains in the rubber network are ductile when the dense domains are dispersed in the rubber, which does not degrade the elasticity or other mechanical properties. Thus, inhomogeneous cross-linked networks can reinforce and toughen rubber materials, with long chains in the sparsely cross-linked domains contributing to the high elongation at break and short chains in the densely cross-linked domains contributing to the high modulus.

**Figure 9:**
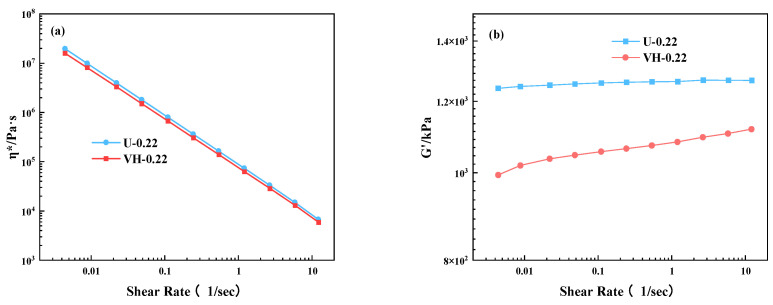
Frequency scanning curves of MVQ rubbers: (**a**) complex viscosity (η*) and (**b**) storage modulus (G′) versus frequency.

In addition, Table 4 shows that the mechanical properties of the U-0.22 and VH-0.22 samples differ markedly. Therefore, rheological analysis was conducted on these two samples with the expectation that the rheological data could reflect the differences in their molecular structure. Figure 9 shows the rheological properties of the two samples with the same pre-curing time (3 min). The complex viscosity (η*)) and storage modulus (G′) of MVQ rubbers tended to increase with increasing cross-link density over the entire frequency range [16]. As shown in Figure 9, the cross-link density of the U-0.22 sample is high, and the three-dimensional network structure formed inside the silicone rubber is complete. The storage modulus (G′)) shows a ‘plateau zone’, indicating the formation of a cross-linked network structure inside the sample. Figure 9b shows that the G′ of the U-0.22 sample exhibits a more obvious ‘plateau zone’, indicating that its cross-linked network is more ordered and homogeneous. According to the theory of rubber elasticity [34,35], the extension of molecular chain segments is limited, and the strain at the breaking point depends on the molecular weight ($M_{c}$) [36,37]. The higher cross-link density results in a lower $M_{c}$ between the cross-linking sites, allowing for an earlier initial fracture stress. As a result, the average cross-linked network structure of the U-0.22 sample is more ordered and even under the same vinyl content, which limits the movement of the molecular chains. Hence, the mechanical properties are poorer.

### 3.6. Friction Properties Analysis

Friction characteristics are important parameters for measuring the performance of rubber materials and an important factor to consider for the practical application of rubber sealing products. Rubber materials have a low modulus due to their entropic elasticity. With a lower modulus, the ability to produce deformation is greater. When contacting another surface under a certain normal load, the material with a low modulus can produce greater deformation, increasing the actual contact with the surface with greater adhesion. Therefore, the coefficient of friction strongly correlates to the elastic modulus, which can be confirmed in the previous literature [38,39].

The elastic modulus of the rubber material can be expressed in terms of the number-averaged molecular weight (*M_c_*). The molecular chains in the rubber network shorten as the degree of cross-linking increases. However, the elastic modulus is equivalent to the shear modulus *G* [40], which can be easily derived as follows:

(5) $$G=\frac{\rho RT}{M_{c}}$$

where ρ is the density of the rubber, $M_{c}$ is the number-average molecular weight between adjacent cross-linked bonds, R is the molar gas constant, and T is the thermodynamic temperature. The cross-link density $\upsilon_{c}$ is measured in mol/cm^3^. The relationship between the cross-link density $\upsilon_{c}$ and the number-average molecular weight (*M_c_*) of the vulcanized MVQ samples can be expressed as follows [41]:

(6) $$\upsilon_{c}\approx \frac{1}{{2\times M}_{c}}$$

**Figure 10:**
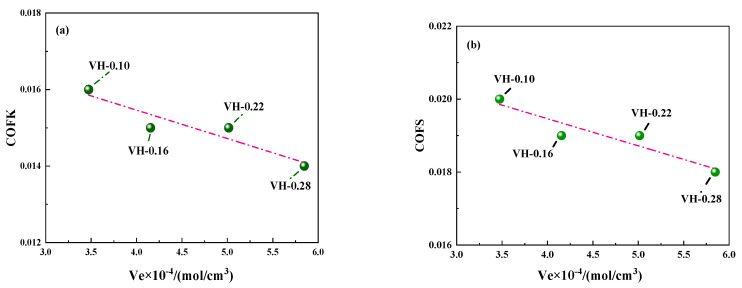
(**a**) Coefficient of kinetic friction (COFK) versus cross-link density and (**b**) coefficient of static friction (COFS) versus cross-link density.

Based on Equations (5) and (6), a higher cross-link density leads to a smaller molecular weight between adjacent cross-linking bonds, a higher shear modulus, and a smaller coefficient of friction. Therefore, cross-link density variation significantly affects the coefficient of friction. Figure 10 shows the friction coefficient curve of the composites versus cross-link density. The dynamic and static friction coefficients of the VH samples decrease slightly with the increase in cross-link density, consistent with the modulus trend in Figure 8.

### 3.7. Filler Networks and Dispersion Morphology

In nanocomposites, the strength of the filler network structure is often indirectly reflected by the Payne effect [42], a phenomenon proposed by Payne in 1962. With cyclic shear stress applied to a nanofiller/elastomer complex with increasing strain, the storage modulus (G′) of the material decreases rapidly when the strain increases to a critical point [43]. The difference between the maximum and minimum storage modulus (ΔG′) reflects the strength of the Payne effect.

**Figure 11:**
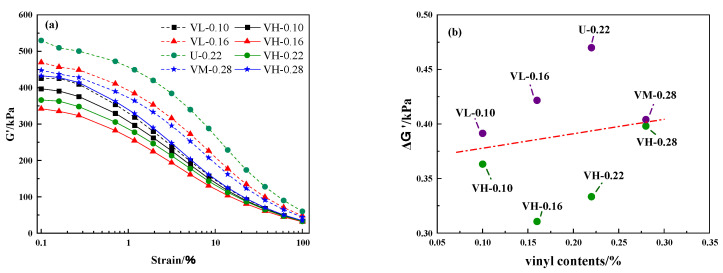
(**a**) Scanning curves of storage modulus G′ versus strain for MVQ rubbers and (**b**) vinyl content curve versus ΔG′ for MVQ rubbers.

A larger ΔG′ indicates a stronger Payne effect in the material. The Payne effect is widely considered to be caused by the disruption of the rigid filler network structure [5,44]. Based on this, a stronger Payne effect implies a stronger filler network structure, while a stronger filler network structure results in a poorer dispersion of nanofillers. The storage modulus G′-strain scanning curve is shown in Figure 11a, and the relationship between ΔG′ and the vinyl content can be observed in Figure 11b. Under the same vinyl content, the ΔG′ of the VH samples is lower than that of the other samples, indicating better filler network formation in the matrix of the other samples. However, the silica dispersion is poor, leading to its poor reinforcing effect.

**Figure 12:**
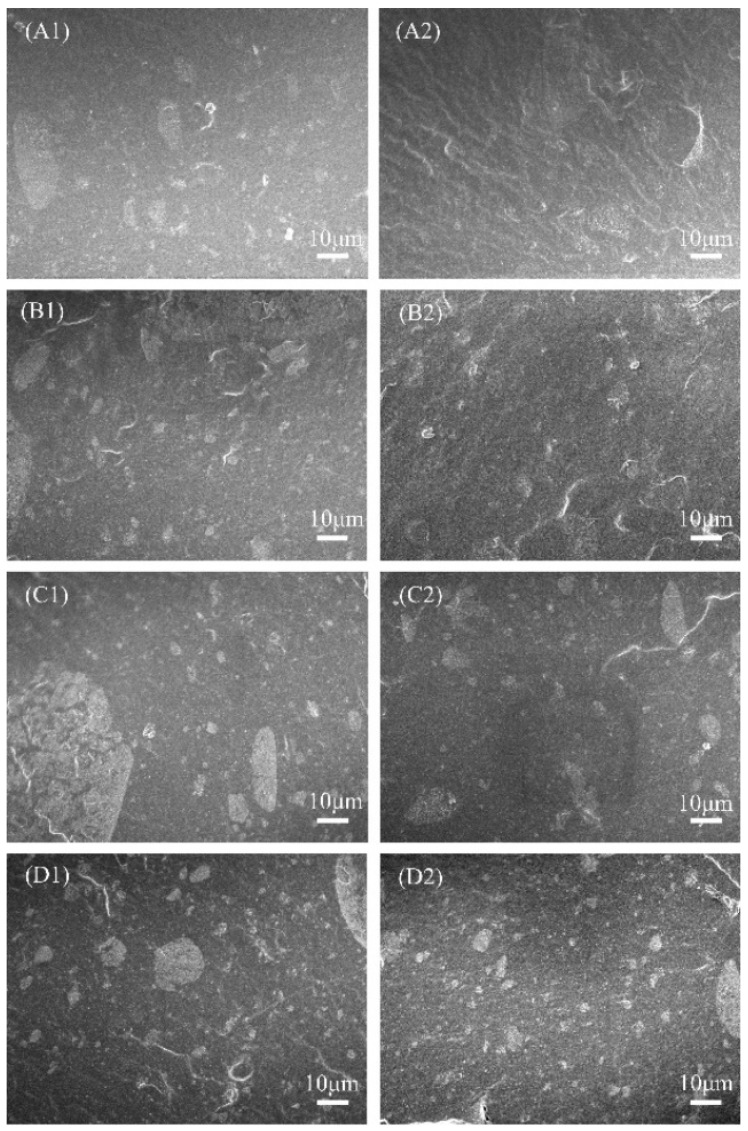
SEM images of different MVQ composites: (**A1**) VL-0.10; (**A2**) VH-0.10, (**B1**) VL-0.16, (**B2**) VH-0.16, (**C1**) U-0.22, (**C2**) VH-0.22, (**D1**) VM-0.28, and (**D2**) VH-0.28.

Silica filler dispersion inside the VMQ rubbers was observed via SEM, and the results are shown in Figure 12. As an important reinforcing filler, the dispersion of silica particles in the rubber matrix directly affects the mechanical properties. The dark part in Figure 12 is the rubber matrix, and the irregular white particles are silica fillers. Under the same magnification, the silica distribution in the VH samples is more uniform with smaller agglomerations. This also explains the much better mechanical properties of the VH samples than those of the U, VL, and VM samples with similar vinyl content.

## 4. Conclusions

This study investigated the effect of vinyl content on the molecular structure of raw MVQ rubbers and the mechanical properties of MVQ composites. The molecular structures of raw MVQ rubbers with different vinyl contents were studied, and the results showed that the MVQ with high vinyl content is a branched polymer compared with the MVQ with low vinyl content. The vinyl group on the side chain is one of the main cross-linking reaction sites, and when MVQs with different vinyl contents are mixed, the distribution of vinyl groups within the composites is not uniform, and an inhomogeneous cross-linking network can be obtained.

In addition, the Flory–Rehner model and Mooney–Rivlin curves showed that long chains in the inhomogeneous network favoured high elongation at break and short chains favoured high modulus. The inhomogeneous network enabled the silicone rubber composites to maintain both good flexibility and high strength. The Payne effect and scanning electron microscopy images showed that the inhomogeneous network improved the dispersion of silica in silicone rubber, and the improvement of mechanical properties was directly related to the inhomogeneous network. In summary, the introduction of an inhomogeneous network into the rubber network can effectively improve the mechanical properties of the material, which can achieve the regulation of the microstructure and macroscopic properties of silicone rubber.

## Figures and Tables

**Table 1:** Compound formulations.

Samples	Different Vinyl Contents			
	MVQ-0.08%/phr	MVQ-0.22%/phr	MVQ-3%/phr	Total
VL-0.10	85.7	14.3		0.10
VH-0.10	99.3		0.7	0.10
VL-0.16	42.8	57.2		0.16
VH-0.16	97.2		2.8	0.16
U-0.22		100		0.22
VH-0.22	95.2		4.8	0.22
VH-0.28	93.2		6.8	0.28
VM-0.28		97.8	2.2	0.28

Note: Silicone rubber is 100 phr, hydroxyl silicone oil is 4 phr, fumed silica is 40 phr, and DBPMH is 1.5 phr. Sample VH: two MVQ rubbers used in a blend with the largest difference in vinyl molar content (molar content of 3% and 0.08%); Sample VM: two silicone rubbers with a medium difference in vinyl content (content of 3% and 0.22%); Sample VL: the smallest difference in vinyl content (0.22% and 0.08%); Sample U: a single vinyl content silicone rubber formulated with a homogeneous cross-linked network.

**Table 2:** LCB indexes of MVQ rubbers with different vinyl contents.

Samples	LCB Indexes	Lissajous Curves
VMQ-0.08%	−0.37	Secondary double rings
VMQ-0.22%	−0.05	Secondary double rings
VMQ-3%	0.19	No secondary double rings

**Table 3:** Cross-link density measured using the equilibrium swelling method and the Mooney–Rivlin curve.

Samples	*υ_c_* × 10^−4^ (mol·cm^−3^) (Swelling)	*υ_c_* × 10^−4^ (mol·cm^−3^) (Mooney–Rivlin)
VL-0.10	3.562	0.692
VH-0.10	3.473	0.408
VL-0.16	4.779	1.712
VH-0.16	4.154	1.291
U-0.22	6.106	2.409
VH-0.22	5.015	1.735
VM-0.28	6.468	2.561
VH-0.28	5.848	2.059

**Table 4:** The mechanical properties of the samples.

Samples	Tensile Strength (MPa)	Tearing Strength (kN/mm)	Elongation at Break (%)	Hardness
VL-0.10	4.881	14.06	319.83	54
VH-0.10	5.568	15.91	353.23	52
VL-0.16	5.329	14.33	239.67	60
VH-0.16	5.393	16.04	321.79	56
U-0.22	3.536	12.76	115.14	64
VH-0.22	5.277	17.05	302.53	59
VM-0.28	4.839	13.12	168.35	65
VH-0.28	5.144	18.34	232.34	62

## Data Availability

Data is contained within the article.
